# Severe achondroplasia due to two de novo variants in the transmembrane domain of *FGFR3* on the same allele: A case report

**DOI:** 10.1002/mgg3.1148

**Published:** 2020-01-23

**Authors:** Tadashi Nagata, Masaki Matsushita, Kenichi Mishima, Yasunari Kamiya, Kohji Kato, Miho Toyama, Tomoo Ogi, Naoki Ishiguro, Hiroshi Kitoh

**Affiliations:** ^1^ Department of Orthopaedic Surgery Nagoya University Graduate School of Medicine Nagoya Japan; ^2^ Department of Genetics Research Institute of Environmental Medicine (RIeM) Nagoya University Nagoya Japan; ^3^ Department of Orthopaedic Surgery Aichi Children's Health and Medical Center Obu Japan

**Keywords:** achondroplasia, allele‐specific PCR, exome sequencing, FGFR3, skeletal dysplasia

## Abstract

**Background:**

Achondroplasia (ACH), the most common form of short‐limbed skeletal dysplasia, is caused by gain‐of‐function mutations in the fibroblast growth factor receptor 3 (*FGFR3*) gene. More than 97% of patients result from a heterozygous p.G380R mutation in the *FGFR3* gene. We present here a child who had two de novo variants in the *FGFR3* on the same allele, a common p.G380R mutation and a novel p.S378N variant.

**Methods:**

A 3‐year‐old Japanese girl born from non‐consanguineous healthy parents showed more severe clinical and radiological phenotypes than classic ACH, including severe short‐limbed short stature with marked ossification defects in the metaphysis and epiphysis, hydrocephalus and cervicomedullary compression due to foramen magnum stenosis, prolonged pulmonary hypoplasia, and significant delay in the gross motor development. Genomic DNA was extracted from the proband and whole‐exome sequencing was performed. The variants were subsequently confirmed by Sanger sequencing.

**Results:**

Mutation analysis demonstrated that the proband had p.S378N (c.1133G>A) and p.G380R (c.1138G>A) variants in the *FGFR3* gene. Both variants were not detected in her parents and therefore considered de novo. An allele‐specific PCR was developed in order to determine whether these mutations were on the same allele (cis) or on different alleles (trans). The c.1138G>A mutation was found in the PCR product generated with the primer for the mutant 1133A, but it was not detected in the product with the wild‐type 1133G, confirming that p.S378N and p.G380R variants were located on the same allele (cis).

**Conclusion:**

This is the second case who had two *FGFR3* variants in the transmembrane domain on the same allele. The p.S378N variant may provide an additive effect on the activating receptor with the p.G380R mutation and alter the protein function, which could be responsible for the severe phenotype of the present case.

## Graphical abstract

We present a case who had two de novo variants in the FGFR3 on the same allele, a common p.G380R mutation and a novel p.S378N variant. The p.S378N variant may provide an additive effect on the activating receptor with the p.G380R mutation and alter the protein function, which could be responsible for severe phenotype of the present case.

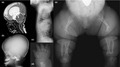

## 1 INTRODUCTION

Achondroplasia (ACH) is the most common form of short‐limbed skeletal dysplasia, with an estimated prevalence of one in 16,000–26,000 live births (Waller et al., 2008). ACH is caused by activating mutations in the fibroblast growth factor receptor 3 (*FGFR3*) gene, which is a negative regulator of longitudinal bone growth (Rousseau et al., 1994; Shiang et al., 1994). Approximately 98% of patients harbor a heterozygous p.G380R mutation that maps to the transmembrane domain of the *FGFR3* gene (Bonaventure et al., 1996). Infants with ACH, thus, present relatively homogeneous skeletal features with macrocephaly with frontal bossing, midface hypoplasia, increased lumbar lordosis, limitation of elbow extension, and trident hands (Horton, Hall, & Hecht, 2007). Recently, phase 2 clinical trial using vosoritide, which is a biological analog of C‐type natriuretic peptide, demonstrated increased growth velocity with minimal side effects in children with ACH (Savarirayan et al., 2019).

Homozygous p.G380R mutations lead to a lethal skeletal phenotype with a narrow thoracic cage, neurologic deficits from cervicomedullary stenosis, and stillbirth due to severe respiratory insufficiency (Aterman, Welch, & Taylor, 1983). Several patients with two different *FGFR3* mutations have been reported. The majority of these patients were compound heterozygous for the classic ACH mutation (p.G380R) and the p.N540K mutation associated with hypochondroplasia (HCH), and they showed a milder skeletal phenotype than homozygous ACH (Chitayat et al., 1999; Flynn & Pauli, 2003; Huggins et al., 1999; Prinos, Costa, Sommer, Kilpatrick, & Tsipouras, 1995; Sommer, Young‐Wee, & Frye, 1987). Generally, these patients inherited both mutations from their affected parents.

Here, we report on a Japanese girl manifesting a more severe skeletal dysplasia than classic ACH, with two de novo *FGFR3* variants, the common p.G380R mutation and the p.S378N variant, both of which were located on the same allele. To the best of our knowledge, this is the second case with two *FGFR3* mutations on the same allele.

## 2 CLINICAL REPORT

**Figure 1:**
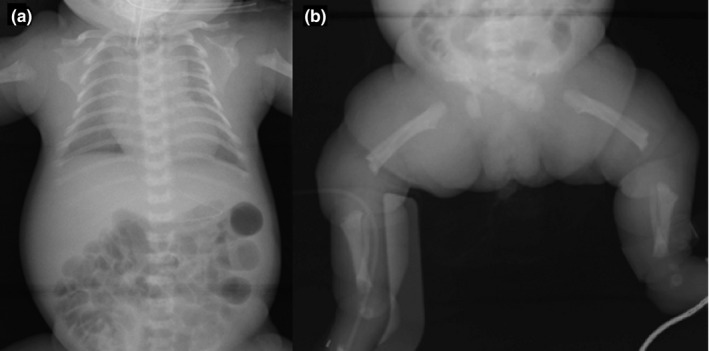
Neonatal radiographs. Anteroposterior radiograph of the thorax and vertebrae demonstrated narrow rib cage, platyspondyly, and narrow interpediculate distance of the lumbar spine (a). Anteroposterior radiograph of the pelvis and lower limb showed squared iliac wings, narrow sacrosciatic notches, shortened long bones with metaphyseal irregularities, proximal femoral translucencies, and bowed fibulae (b)

The proband, a 3‐year‐old girl, was the first child of non‐consanguineous Japanese parents. Their younger sister was normal. Screening ultrasound at 33 weeks of trimester showed increased biparietal diameter (85.9 mm: +1.2 *SD*) and shortening of the femur length (40.9 mm: −5.8 *SD*). The infant was delivered at 37 weeks of gestation with a birth weight of 2.690 g (+0.3 *SD*), length of 40.0 cm (−3.1 *SD*), and head circumference of 33.6 cm (+0.6 *SD*), from a 33‐year‐old mother and a 34‐year‐old father. The Apgar scores were four and six at 1 and 5 min, respectively. She showed severe shortening of the upper and lower extremities with redundant skin folds, a small chest, frontal bossing, midface hypoplasia, and trident hands. Neonatal radiographs showed a small thorax with short ribs, abdominal distension (Figure 1a), hypoplastic ilium with narrow sciatic notch, proximal femoral radiolucencies, and metaphyseal cupping of the long tubular bones (Figure 1b). Based on these clinical and radiological characteristics, she was tentatively diagnosed as ACH.

**Figure 2:**
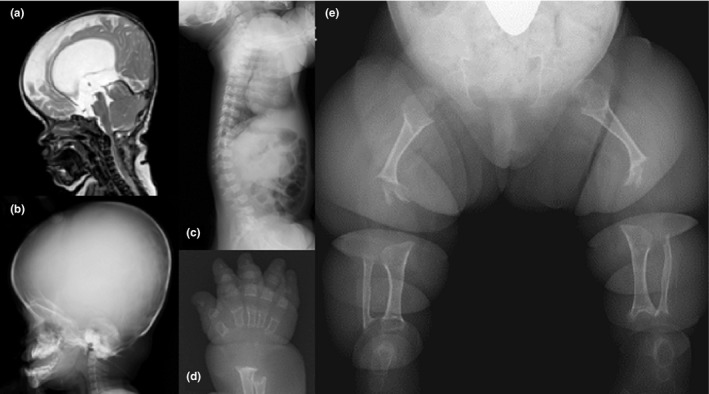
Image findings during infancy. Magnetic resonance imaging of the head and cervical spine at the age of 3 months indicated hydrocephalus and foramen magnum stenosis (a). Lateral radiograph of the skull at the age of 1 year showed a large skull with relatively small cranial base, hypoplastic maxilla, and protruded mandible (b). Lateral radiograph of the spine at the age of 1 year demonstrated mild platyspondyly with tongue‐like protrusion of the lumbar spine and extremely shortened ribs with metaphyseal flaring (c). Anteroposterior radiograph of the right hand at the age of 1 year showed generalized brachydactyly with marked metaphyseal irregularities (d). Anteroposterior radiograph of the lower limbs at the age of 2.7 years demonstrated extremely shortened and thin long tubular bones with deficient ossifications at the metaphysis and epiphysis (e)

**Figure 3:**
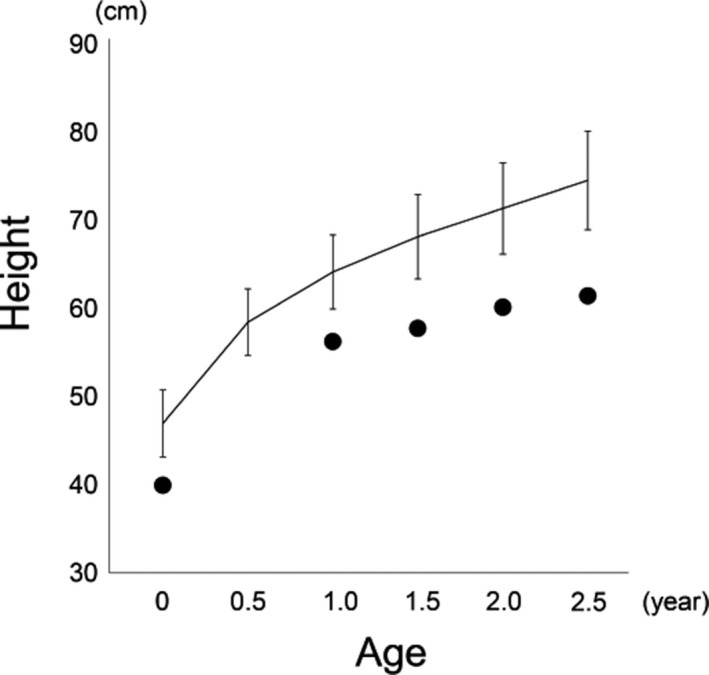
Growth curves of the proband and standard Achondroplasia (ACH) patients. The dots and line showed the height of proband and the mean ± 2 *SD* of standard ACH patients (Tachibana et al., 1997), respectively

She showed evident signs of respiratory distress and required intubation with an artificial respirator at a neonatal intensive care unit. Craniocervical magnetic resonance imaging (MRI) demonstrated hydrocephalus and foramen magnum stenosis with cervicomedullary compression (Figure 2a). She underwent a surgical decompression of craniocervical junction at the age of 3 months for central respiratory insufficiency. However, she needed all‐day continuous positive airway pressure (CPAP) until 6 months of age and night‐time CPAP, thereafter, due to persistent pulmonary hypoplasia. In addition to respiratory problems, the linear growth was severely delayed with a weight of 6.2 kg (−2.5 *SD*) and a height of 56.3 cm (−6.8 *SD*) at 1 year of age, and a weight of 8.8 kg (−2.6 *SD*) and a height of 64.7 cm (−7.8 *SD*) at 2 year 9 months of age. According to the growth chart of Japanese ACH children (Tachibana, Suwa, Nishiyama, & Matsuda, 1997), her height was −3.7 *SD* at 1 year of age and −4.6 *SD* at 2 year 9 months of age (Figure 3). Her gross motor development was markedly delayed, as she was not sitting independently at 3 years of age. Cognitive and language development were also delayed.

Craniofacial dysmorphisms including frontal bossing, hypoplastic maxilla, and short cranial base were remarkable (Figure 2b). Thoracolumbar kyphosis and mild platyspondyly with central tongue‐like protrusions were observed on the lateral spine radiograph (Figure 2c). Brachydactyly with metaphyseal cupping of phalangeal bones was severe (Figure 2d). In the long tubular bones of the lower limbs, there were not only shortening but also poor epiphyseal and metaphyseal ossifications, proximal femoral radiolucencies, and slender diaphysis (Figure 2e).

## 3 MUTATION ANALYSIS

**Figure 4:**
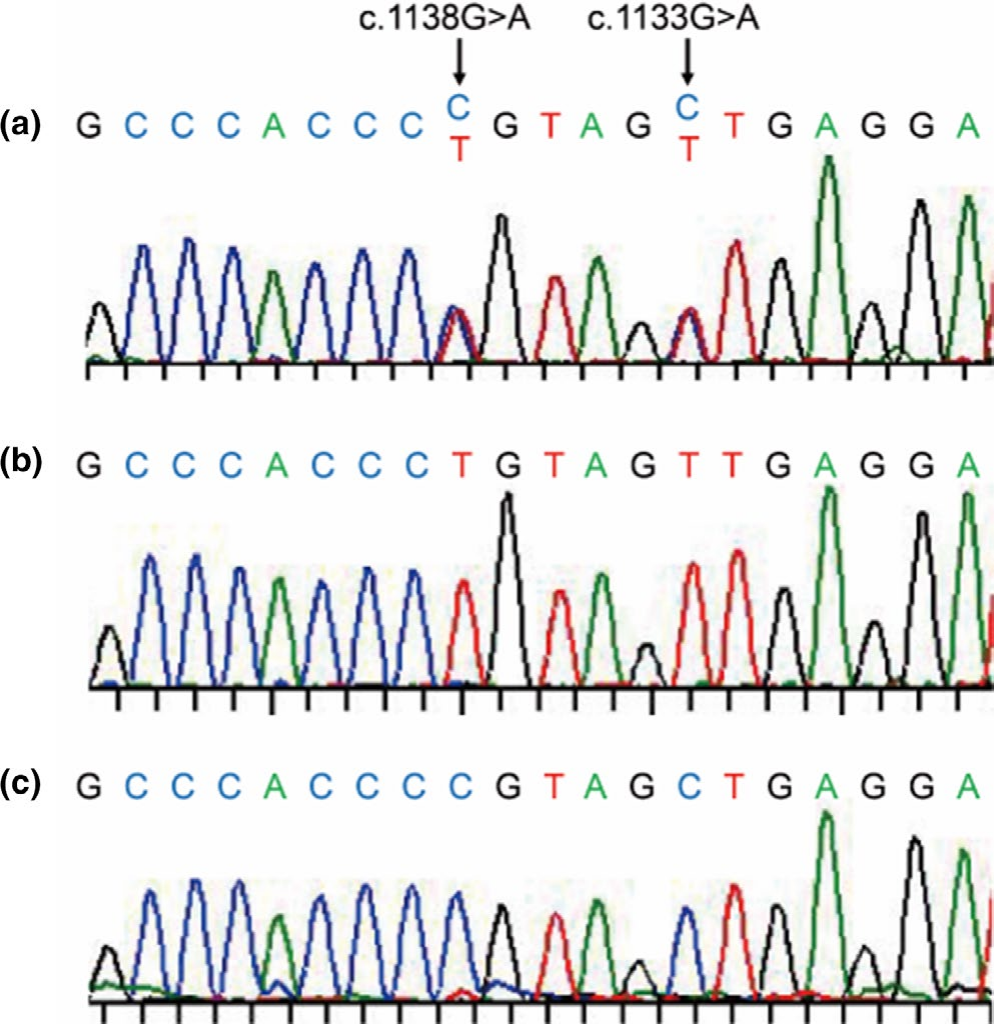
Sanger sequencing of the proband in the region about the mutations in the FGFR3 gene. There were two heterozygous mutations of the c.1133G>A and the c.1138G>A (a). The allele‐specific PCR revealed that the c.1133G>A and the c.1138G>A were in the same allele (b), while no mutations were detected in another allele (c)

After informed consent was obtained from her parents, genomic DNAs were purified from peripheral blood of the proband and saliva of her parents using the QIAamp DNA Blood Midi kit (Qiagen, Inc.). Considering the possibility of other skeletal dysplasia than ACH from her severe phenotype, whole‐exome sequencing was performed on the proband using the Sure Select v.6 whole exon target enrichment system (Agilent) and HiSeq2500 platform (Illumina). Variant annotation revealed that the proband had p.S378N (c.1133G>A) and p.G380R (c.1138G>A) in the *FGFR3* gene. Next, we performed Sanger sequencing using the forward primer (5'‐cctcaacgcccatgtcttt‐3') and the reverse primer (5'‐aggcagctcagaacctggta‐3') located in exon‐intron boundaries using the Applied Biosystems 3730xl DNA Analyzer (Thermo Fisher Scientific). The two *FGFR3* variants were confirmed in the proband (Figure 4a), while there were no mutations in her parents.

The alternative forward primers for the allele‐specific PCR were created with 3'‐ends overlapping position 1133 of the *FGFR3* sequence (5'‐tgtgtatgcaggcatcctcag‐3' for wild‐type 1133G and 5'‐tgtgtatgcaggcatcctcaa‐3' for mutant 1133A). The PCR products were obtained by these alternative forward primers and the reverse primer (5'‐aggcagctcagaacctggta‐3') in the exon‐intron boundary with sequencing by the reverse primer. The c.1138G>A mutation was found in the PCR product generated with the primer for the mutant 1133A (Figure 4b), but it was not detected in the product with wild‐type 1133G (Figure 4c), confirming that p.S378N and p.G380R variants were located on the same allele.

## 4 DISCUSSION

Here, we report on a child who had p.S378N and p.G380R variants on the same allele in the *FGFR3* gene. Clinical and radiological findings of the current subject were more severe than classic ACH children but milder than homozygous ACH or thanatophoric dysplasia. Actually, she could survive irrespective of having moderate respiratory distress, but her height was apparently shorter than the standard of ACH children. Shortening was more marked in the lower limbs rather than in the trunk, with distinct findings of secondary ossification defects. In addition to epi‐metaphyseal involvements, the shafts of the long tubular bones were extremely thin, suggesting the impairment of intramembranous ossification. The motor milestones of the current subject were too delayed compared to those of standard ACH children, which are usually delayed due to muscular hypotonia (Trotter, Hall, & American Academy of Pediatrics Committee on Genetics, 2005). Moreover, she had severe complications, including hydrocephalus, pulmonary hypoplasia, and cervicomedullary compression. Rump et al. (Rump et al., 2006) reported a severe ACH infant with a similar degree of severity and complications to our subject, who had p.L377R and p.G380R variants on the same allele. Although skeletal phenotypes were more severe in our subject, these two children may have the same molecular mechanism of *FGFR3* activation.

The *FGFR3* receptor with p.G380R was activated via a delay in the down‐regulation of the receptor (Monsonego‐Ornan, Adar, Feferman, Segev, & Yayon, 2000). Patients with this common mutation show relatively homogeneous skeletal phenotypes, although the rate of associated complications varies. Since there have been no case reports describing the patients who had the p.L377R or the p.S378N variant independently, we do not know whether these variants are pathogenic or not. A previous in vitro study by ToxR plasmids carrying the *FGFR3* transmembrane domain demonstrated that the substitutions of the p.S378 to glycine, alanine, cysteine, or isoleucine showed no or very little changes in the dimerization of the receptor (Mudumbi, Julius, Herrmann, & Li, 2013), although the effect of the p.S378N was not examined. They also demonstrated that p.S378I significantly altered the dimerization propensity of the receptor with the p.A391E, which is a pathogenic mutation causing Crouzon syndrome with acanthosis nigricans. Considering a severe phenotype of the current subject, we speculate that the p.S378N may provide an additive effect on the activating receptor with the p.G380R mutation and alter the protein function.

## CONFLICT OF INTEREST

The authors have no conflicts of interest to declare.
